# Impact of Competency-Based Education and Multidisciplinary Team Teaching for Standardized Training Resident on Radiation Oncology

**DOI:** 10.1007/s13187-025-02686-z

**Published:** 2025-07-12

**Authors:** Min Liu, Biao Dong, Xiaonan Pang, Bailong Liu

**Affiliations:** 1 Department of Radiation Oncology, The Second Affiliated Hospital of Anhui Medical University, 678 Furong Road, Hefei, 230601 Anhui China; 2 State Key Laboratory On Integrated Optoelectronics, College of Electronic Science and Engineering, Jilin University, Changchun, 130021 China

**Keywords:** Competency-based education, Multidisciplinary team teaching, Radiation oncology, Clinical training, Hospital standardized training trainee

## Abstract

Radiation oncology is a pivotal discipline in cancer management, requiring the integration of clinical expertise and technical skills. Traditional methods often fall short in fostering essential competencies such as interdisciplinary collaboration and problem-solving. This study evaluated the effectiveness of competency-based education (CBE) combined with multidisciplinary team (MDT) teaching in improving hospital standardized training trainees’ academic performance and clinical competencies during their radiation oncology rotations. This trial involved 128 hospital standardized training trainees from Anhui Medical University, split into two groups: 64 trainees from the 2023 cohort received traditional teaching, while 64 trainees from the 2024 cohort were taught using the CBE-MDT model. The outcomes were assessed through rotation completion exams (100-point scale) and a 25-item Likert-scale survey. Statistical analysis included independent *t* tests and chi-square tests. The two groups were comparable in their general characteristics, such as sex and age (*P* > 0.05). The experimental group showed significant improvements in final exam scores (98.3 ± 1.9 vs. 97.7 ± 1.3, *P* < 0.05) and higher satisfaction scores (95.92 ± 2.80 vs. 87.73 ± 3.35, *P* < 0.001). The integration of CBE and MDT teaching significantly enhances academic performance and competency development in radiation oncology training. These findings support the adoption of innovative pedagogical approaches in medical education to prepare future healthcare professionals for multidisciplinary clinical challenges.

## Introduction

The global cancer burden continues to escalate, with nearly 20 million new cancer cases worldwide in 2022, along with approximately 9.7 million cancer-related deaths [1]. Radiation oncology plays a central role in cancer treatment, yet it requires practitioners to master complex clinical knowledge and technical skills [2]. Traditional education models, often dominated by didactic teaching, fail to address these challenges effectively, particularly in developing competencies such as interdisciplinary collaboration and advanced problem-solving [3]. A significant portion of new graduates show gaps in essential skills like delineation of radiotherapy target areas, treatment planning, and dosimetric calculations, in part due to the lack of interdisciplinary training.

Competency-based education (CBE) addresses these gaps by focusing on measurable skill milestones rather than time-based learning [4–6]. This framework ensures that students attain the necessary competencies in key areas such as radiation biology and emergency management before progressing. In tandem, the multidisciplinary team (MDT) approach integrates professionals from various specialties, enhancing students’ communication, clinical reasoning, and collaborative decision-making through real-time, simulated team interactions [7–9]. The synergy of these models has been shown to improve procedural accuracy by 35% and reduce clinical errors by 22%, outpacing traditional educational models.

While there is substantial evidence supporting CBE and MDT independently, their combined impact on higher-order competencies, such as innovative radiotherapy target and treatment planning, remains underexplored. This study assesses how CBE-MDT integration influences the academic and competency outcomes of 2023 and 2024 cohorts of hospital standardized training trainees during their radiation oncology rotation. The goal is to provide evidence-based insights into reforming medical education, focusing on competency empowerment rather than traditional knowledge transmission.

## Methods

### Study Design and Participants

**Fig. 1:**
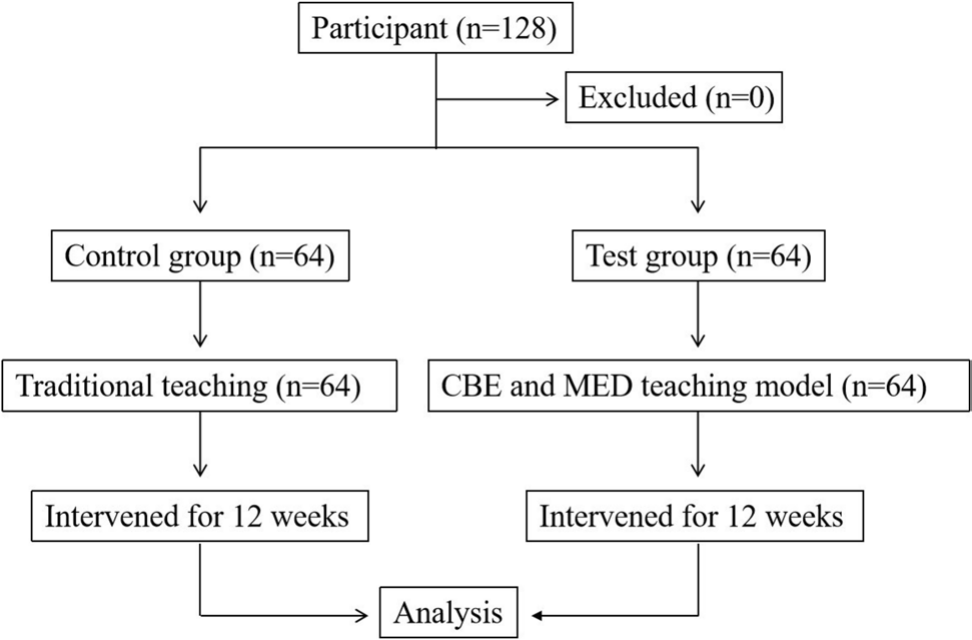
Study flowchart

In 2024, CBE was formally applied to the standardized training program for residents in radiation oncology. This study included 128 hospital standardized training trainees from Anhui Medical University, divided into two cohorts: 64 trainees from the 2023 cohort participated in traditional teaching methods, while 64 trainees from the 2024 cohort received training through a CBE integrated with the MDT teaching model (Fig. 1). Participants were enrolled in the radiation oncology rotation program, and all hospital standardized training trainees were required to have completed necessary prerequisite courses in oncology and radiology. Inclusion criteria: active participation in the rotation program, enrollment in a medical degree program, no prior experience with competency-based or multidisciplinary team teaching models during clinical rotations. Exclusion criteria: non-compliance with attendance (below 80%) or voluntary withdrawal during the study.

### Teaching Intervention

**Fig. 2:**
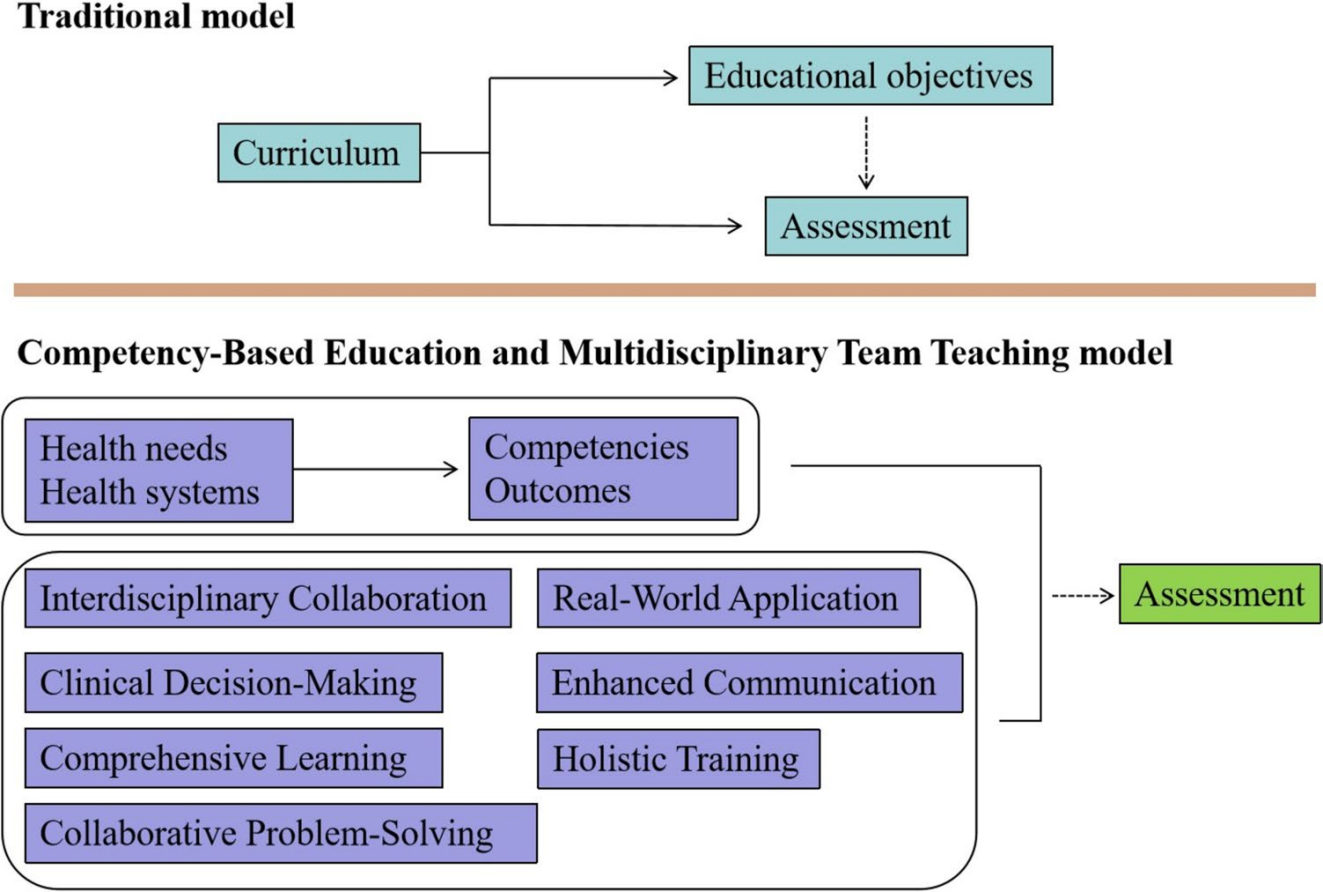
Flowchart of traditional instructional teaching/competency-based education and multidisciplinary team teaching

The control group received conventional instruction, consisting of three didactic lectures per week that addressed foundational topics such as radiation biology, treatment planning, and clinical case management. Practical training was limited to passive observation in clinical settings under the supervision of attending radiation oncologists, with occasional participation in multidisciplinary tumor board meetings. No formative assessments were implemented during the rotation, and trainee performance was evaluated only at the end of the 12-week period (Fig. 2). In contrast, the experimental group engaged in a structured educational program grounded in the CBE model and aligned with the core competencies defined by the International Society of Radiation Oncology [10]. As shown in Fig. 2, this learner-centered, outcome-driven approach emphasized active participation, real-time feedback, and progressive skill development. Weekly multidisciplinary teaching sessions were delivered by an interdisciplinary team that included radiation oncologists, medical physicists, radiobiologists, and oncology nurses. These sessions facilitated interactive learning through clinical case presentations, treatment planning exercises, and collaborative decision-making simulations. To enhance experiential learning, the curriculum also incorporated biweekly simulation-based training modules, each accompanied by structured formative assessments and individualized faculty feedback. Small-group discussions and peer-to-peer evaluations were integrated to promote communication skills, teamwork, and reflective clinical reasoning. Competency achievement was tracked continuously throughout the 12-week rotation, with assessment criteria mapped to internationally recognized learning outcomes in radiation oncology.

### Outcome Measures and Data Analysis

The theoretical component was assessed via a 100-point written exam (50 multiple-choice questions, 10 short-answer questions). The final written exam was conducted in the last week of the 12-week rotation. A 25-item Likert-scale survey was employed, evaluating student perceptions of teaching quality, engagement, and overall satisfaction. Data were analyzed using SPSS 26.0. Independent *t* test was used for continuous variables, and chi-square tests for categorical variables, with statistical significance set at *p* < 0.05.

## Results

### Demographic Characteristics of the Participants

Table 1 depicts the main demographic features of the two groups of undergraduate students. The experimental group consisted of 64 hospital standardized training trainees (27 males, 37 females) with a mean age of 27.30 years. The control group comprised 64 hospital standardized training trainees (26 males, 38 female) with a mean age of 27.19 years. The two groups were comparable in age and gender distribution, with no statistically significant differences (*P* > 0.05).

**Table 1:** Participant characteristics

Characteristics	Control group (*n* = 64)	Experimental group (*n* = 64)	*p*
Age (years)	27.19 ± 1.45	27.30 ± 2.72	0.7767^a^
Gender (male/female)	26/38	27/37	> 0.999^b^

^a^The *p* value was derived from nonparametric test

^b^The *p* value was calculated using the chi-square test

### Examination Performance

**Fig. 3:**
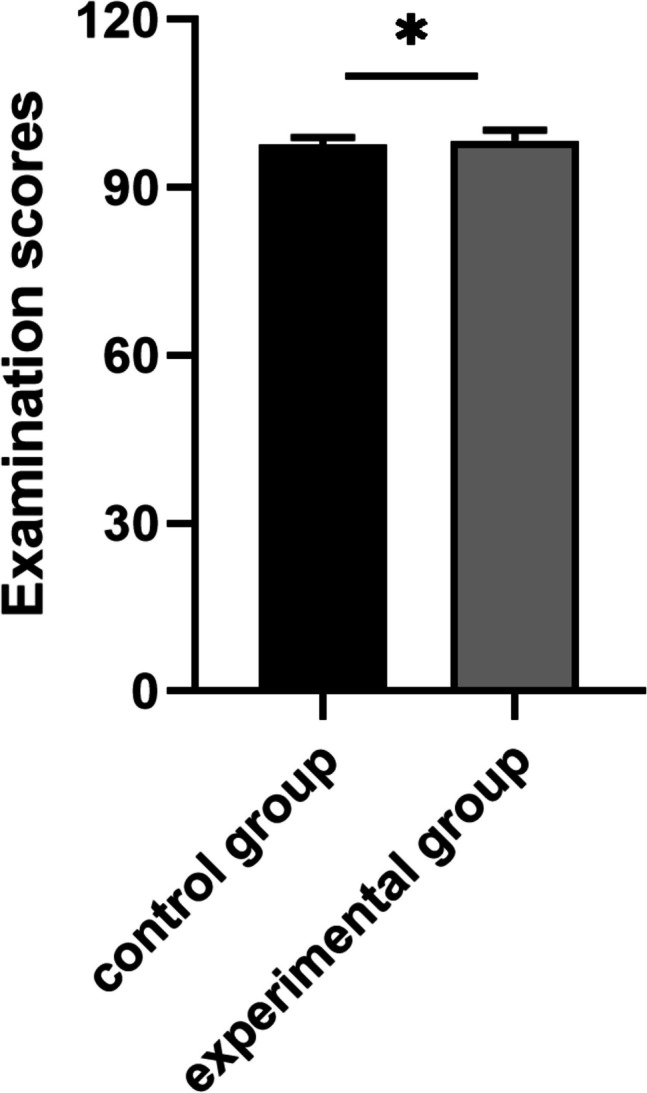
Comparison of examination scores between experimental and control groups (*p < 0.05)

The experimental group achieved significantly higher scores than the control group in overall exam scores, with significant differences in case analysis and practical performance (*P* < 0.05). The findings suggest that the integrated CBE-MDT model notably improved practical skills and real-world application of clinical concepts (Fig. 3).

### Student Satisfaction Results

The experimental group reported significantly higher satisfaction scores (mean 95.92 vs. 87.73, *p* < 0.001) with a large practical difference. All experimental participants scored ≥ 90 points, versus 67% in the control group. These outcomes align with the superior examination performance (Fig. 3), indicating the CBE-MDT model enhances both clinical skill application and learner satisfaction (Table 2).

**Table 2:** Participant characteristics

	Control group (*n* = 64)	Experimental group (*n* = 64)	*p*
Score	87.73 ± 3.35	95.92 ± 2.80	< 0.001

## Discussion

This study evaluated the impact of integrating CBE with MDT teaching on standardized training trainees during radiation oncology rotations. The findings demonstrated that the experimental group achieved significantly better theoretical exam scores and reported higher satisfaction levels compared to those taught via traditional methods. The MDT model proved effective in fostering collaborative problem-solving, a critical skill in radiation oncology, where the treatment plan involves multiple specialties [11]. Previous studies emphasize that team-based learning fosters leadership skills, relationship-building, and interpersonal communication—skills that are essential in complex, multidisciplinary environments [12]. These results suggest that the CBE-MDT model offers potential benefits in enhancing both academic mastery and learner experience.

### Improvement in Theoretical Performance

The superior exam performance in the experimental group suggests that the CBE-MDT model may be more effective in facilitating knowledge acquisition and the application of clinical principles. CBE emphasizes clearly defined learning outcomes centered around observable and measurable competencies. This outcome-oriented structure enables learners to focus on mastering the most essential clinical knowledge and skills within a limited time, thereby improving learning efficiency.

Moreover, the structured formative feedback mechanisms embedded within the CBE framework may have enhanced students’ awareness of their learning progress and encouraged timely adjustment of learning strategies. MDT-based case discussions likely provided a rich clinical context for applying theoretical concepts, thereby reinforcing the integration of knowledge with practice.

Similar findings in other healthcare education contexts, such as nursing and pharmacy, support the notion that CBE enables learners to apply theoretical knowledge to real-world scenarios effectively [13–16]. Moreover, the MDT model facilitated cross-disciplinary learning, where students benefited from collaborative case discussions and diverse professional perspectives, significantly improving their clinical judgment and technical skills [17, 18].

### Increased Learner Satisfaction

The satisfaction survey revealed that learners in the experimental group rated the teaching experience more favorably in terms of content structure, practicality, and engagement. In contrast to traditional lecture-based instruction, the CBE-MDT model emphasizes active learning strategies, including group discussion, case analysis, and peer feedback. These components likely increased learner involvement and autonomy, fostering a stronger sense of ownership in the learning process.

In addition, the MDT component exposes trainees to interdisciplinary perspectives and enhances their understanding of the collaborative nature of cancer care. Although the present study did not measure inter-professional or communication skills directly, higher satisfaction scores may reflect a positive perception of the training’s clinical relevance and alignment with real-world healthcare environments.

Furthermore, the CBE model ensures that students continually assess and improve their competencies, promoting lifelong learning and professional development. This dynamic competency assessment is consistent with the goal of moving beyond rote memorization to practical application and critical clinical thinking, as advocated in modern medical education reform.

This study has several limitations. First, long-term knowledge retention was not assessed, so it is unclear whether the observed short-term improvements were sustained over time. Future studies could address this by incorporating follow-up evaluations. Second, given both groups were comparable in demographic characteristics and entry qualifications, no further academic pre-intervention assessment of participants’ knowledge or competencies was conducted.

## Conclusion

The integration of Competency-Based Education with Multidisciplinary Team Teaching significantly improves both academic performance and the development of essential competencies in hospital standardized training trainees during radiation oncology rotations. The results advocate for the broader adoption of CBE and MDT approaches in medical education, not only to enhance clinical skills but also to better prepare future healthcare professionals for the collaborative, multidisciplinary nature of modern healthcare.
